# Serum Perfluorinated Compound Concentration and Attention Deficit/Hyperactivity Disorder in Children 5–18 Years of Age

**DOI:** 10.1289/ehp.1003538

**Published:** 2011-06-10

**Authors:** Cheryl R. Stein, David A. Savitz

**Affiliations:** 1 Department of Preventive Medicine, Mount Sinai School of Medicine, New York, New York, USA; 2 Department of Community Health, and; 3 Department of Obstetrics and Gynecology, Brown University, Providence, Rhode Island, USA

**Keywords:** adolescent, attention deficit disorders, child, epidemiology, fluorocarbons, perfluorooctanoic acid

## Abstract

Background: Perfluorinated compounds (PFCs) are persistent environmental pollutants. Toxicology studies demonstrate the potential for perfluorooctanoic acid (PFOA) and other PFCs to affect human growth and development. Attention deficit/hyperactivity disorder (ADHD) is a developmental disorder with suspected environmental and genetic etiology.

Objectives: We examined the cross-sectional association between serum PFC concentration and parent or self-report of doctor-diagnosed ADHD with and without current ADHD medication.

Methods: We used data from the C8 Health Project, a 2005–2006 survey in a Mid-Ohio Valley community highly exposed to PFOA through contaminated drinking water, to study non-Hispanic white children 5–18 years of age. Logistic regression models were adjusted for age and sex.

Results: Of the 10,546 eligible children, 12.4% reported ADHD and 5.1% reported ADHD plus ADHD medication use. We observed an inverted J-shaped association between PFOA and ADHD, with a small increase in prevalence for the second quartile of exposure compared with the lowest, and a decrease for the highest versus lowest quartile. The prevalence of ADHD plus medication increased with perfluorohexane sulfonate (PFHxS) levels, with an adjusted odds ratio of 1.59 (95% confidence interval, 1.21–2.08) comparing the highest quartile of exposure to the lowest. We observed a modest association between perfluorooctane sulfonate and ADHD with medication.

Conclusions: The most notable finding for PFOA and ADHD, a reduction in prevalence at the highest exposure level, is unlikely to be causal, perhaps reflecting a spurious finding related to the geographic determination of PFOA exposure in this population or to unmeasured behavioral or physiologic correlates of exposure and outcome. Possible positive associations between other PFCs and ADHD, particularly PFHxS, warrant continued investigation.

Perfluorooctanoic acid (PFOA) is a synthetic chemical that has been used in the manufacture of fluoropolymers since the 1950s [U.S. Environmental Protection Agency (EPA) 2009a] and may also result from the breakdown of a related group of chemicals called fluorinated telomers (U.S. EPA 2009b). Fluoropolymers are used in nonstick cookware and clothing made from waterproof, breathable fabric (U.S. EPA 2009b).

PFOA and other perfluorinated compounds (PFCs) with comparable industrial uses—perfluorooctane sulfonate (PFOS), perfluorohexane sulfonate (PFHxS), and perfluorononanoic acid (PFNA)—are persistent environmental pollutants that have been detected worldwide in both wildlife and humans, with higher exposure closer to urbanized and industrialized regions (Houde et al. 2006). In the U.S. general population, PFOA, PFOS, and PFHxS were detected in all serum samples from the 1999–2000 National Health and Nutrition Examination Survey (NHANES); PFNA was detected in 95% of samples (Calafat et al. 2007a). NHANES 2003–2004 data showed minor reductions in the percentage of samples with detectable levels of PFOA, PFOS, and PFHxS, and the geometric mean concentrations for these three compounds dropped slightly (Calafat et al. 2007b). For PFNA, however, the percentage of serum samples with detectable levels increased, and the geometric mean increased from 0.5 ng/mL to 1.0 ng/mL, between the NHANES waves. PFOA and PFOS, the two most commonly studied PFCs, have been detected in maternal and umbilical cord blood (Inoue et al. 2004; Midasch et al. 2007; Monroy et al. 2008) and breast milk (Kuklenyik et al. 2004; So et al. 2006; Tao et al. 2008a, 2008b; Volkel et al. 2008). The serum elimination half-life for PFOA is estimated at 2.3 years (Bartell et al. 2010) to 4 years (Olsen et al. 2007); for PFOS, 5 years (Olsen et al. 2007); and for PFHxS, 8.5 years (Olsen et al. 2007). A half-life estimate is not available for PFNA.

Toxicology studies highlight the potential for PFCs to affect fetal growth and development (reviewed by Lau et al. 2004, 2007; Olsen et al. 2009). Rat pups prenatally exposed to PFOS show delayed behavioral milestones (Luebker et al. 2005), and delayed task learning has been noted in female pups prenatally exposed to PFOS and maternal restraint (Fuentes et al. 2007). The limited developmental toxicology literature suggests possible adverse effects of PFOA and PFOS on fetal growth and viability and postnatal growth (Butenhoff et al. 2004; Lau et al. 2004).

One epidemiologic study examined developmental milestones in relation to PFC exposure (Fei et al. 2008). In a substudy of the Danish National Birth Cohort (*n* = 1,400), early pregnancy plasma PFOA and PFOS levels were essentially unrelated to motor or mental development through 18 months of age, although there were weak associations between increased PFOS levels and sitting without assistance or using wordlike sounds to indicate wants (Fei et al. 2008). A single study has examined the association between PFC exposure and attention deficit/hyperactivity disorder (ADHD) among children from the 1999–2000 and 2003–2004 NHANES and reported increased odds of disease with higher serum PFC levels (Hoffman et al. 2010).

ADHD is a relatively common neurodevelopmental disorder with suspected environmental and genetic etiology (reviewed by Aguiar et al. 2010; Banerjee et al. 2007; Swanson et al. 2007). The disorder, generally recognized by early school age, is characterized by developmentally inappropriate levels of inattention, hyperactivity, and impulsivity [Centers for Disease Control and Prevention (CDC) 2010]. In the 2007 U.S. National Survey of Children’s Health, the estimate of parent-reported ADHD among children 4–17 years of age was 9.5%; 4.8% reported both diagnosis and medication use (CDC 2010). Although prevalence estimates vary, it is generally accepted that the prevalence of this disorder is rising (Aguiar et al. 2010; CDC 2010; Pastor and Reuben 2008). In contrast, the prevalence of learning disorders among children 6–17 years of age, estimated at 8.7%, has been relatively stable from 1997 to 2006 (Pastor and Reuben 2008). Prenatal exposures to alcohol and cigarettes, as well as childhood exposure to lead, polychlorinated biphenyls (PCBs), and methyl mercury, have all been positively associated with ADHD or ADHD-like behaviors (reviewed by Banerjee et al. 2007; Eubig et al. 2010). PFCs, with their widespread exposure and potential for developmental toxicity, warrant examination as an environmental risk factor for ADHD.

A chemical plant in the Mid-Ohio Valley near Parkersburg, West Virginia, has used PFOA in the manufacture of fluoropolymers since 1951. In 2001, a group of residents from the West Virginia and Ohio communities surrounding the plant filed a class action lawsuit alleging health damage from drinking water supplies drawing on PFOA-contaminated groundwater (Frisbee et al. 2009). Groundwater contamination from the Ohio River and air deposition are believed to be the primary exposure routes for this population (Emmett et al. 2006). The settlement of the class action lawsuit included a baseline survey, the C8 Health Project (C8 is another name for PFOA, denoting its chain of eight carbons) (Frisbee et al. 2009). The C8 Health Project included demographic and health questionnaires and measurement of 10 PFCs in serum. In the present study we used these data to examine the cross-sectional association between serum PFOA, PFOS, PFHxS, and PFNA measurements at C8 Health Project enrollment and report of a diagnosis with ADHD or a learning problem among children 5–18 years of age.

## Methods

*C8 Health Project population.* The C8 Health Project enrolled participants between August 2005 and July 2006. The project’s purpose was to collect health data from members of the class action lawsuit through questionnaires and blood tests, including measurement of PFCs. This community was highly exposed to PFOA, but exposure to other PFCs reflects typical background levels. Individuals were eligible to participate in the C8 Health Project if they could provide documentation proving they had consumed water for at least 1 year between 1950 and 3 December 2004 in a water district supplied by Little Hocking Water Association of Ohio; City of Belpre, Ohio; Tupper Plains–Chester District of Ohio; Village of Pomeroy, Ohio; Lubeck Public Service District of West Virginia; Mason County Public Service District of West Virginia; or private water sources within areas of documented PFOA contamination. Participants provided informed consent and were compensated $400 for completing the questionnaire and providing blood. At the time of enrollment, participants did not know their individual exposure to PFOA. The C8 Health Project collected data on 69,030 people. The total number of people eligible to join the class action lawsuit is unknown. Participation rates based on U.S. Census counts of current residents of the eligible water districts are estimated at around 80% and in some ZIP codes within eligible water districts appear close to 100% (Frisbee et al. 2009). The overall participation rate is likely < 80% because former residents were also eligible to participate. In this population, the strongest predictor of PFOA serum level was current residence in a contaminated water district, with distance to the plant directly affecting PFOA levels (Steenland et al. 2009). Because plant emissions varied over the 50-year contamination period, the relative PFOA exposure levels of the water districts remained the same, such that high-exposure water districts were always more exposed than low-exposure water districts, regardless of the absolute exposure levels. Exposure to other PFCs is not determined by residence in a PFOA-contaminated water district and reflects typical background levels, which likely occur through dietary and consumer product exposures (Vestergren and Cousins 2009). Of the 69,030 C8 Health Project participants, 12,016 were 5–18 years of age at enrollment. Of these children, 11,046 (92%) had serum PFC measurements.

*Measures.* Laboratory analysis (Exygen Research Inc., State College, PA) of PFCs used automated solid-phase extraction combined with reverse-phase high-performance liquid chromatography (Kuklenyik et al. 2004). Four PFCs were detectable in 100% of samples (PFOA, PFOS, PFHxS, PFNA); we included these four in our analyses. We examined the association between PFCs and ADHD using restricted cubic splines (Desquilbet and Mariotti 2010) and determined that quartiles of exposure best captured the nonlinear nature of the PFC–outcome associations. All analyses categorized PFCs into quartiles.

We examined the relation between PFC serum concentrations and a diagnosis of ADHD as reported in the C8 Health Project questionnaire. Participants were asked “Has a doctor or health professional ever told you that you have/had ‘Attention Deficit Disorder’ (ADD or ADHD)?” We additionally constructed a second, more sensitive ADHD definition by combining report of ADHD diagnosis with current use of a medication commonly used to treat ADHD (Braun et al. 2006). Participants were asked to list all current prescription and over-the-counter medications they were currently taking for any reason. Based on guidance from clinical experts, medications considered treatment for ADHD were methylphenidate, dextroamphetamine, mixed amphetamine salts, lisdexamfetamine, dexmethylphenidate, atomoxetine, clonidine, guanfacine, imipramine, nortriptyline, bupropion, and carbamazepine. We included several medications that are prescribed off-label for complicated and recalcitrant cases of ADHD. This decision yielded 23 children who reported a diagnosis with ADHD and use of off-label medications only that would not have been included in the analysis of ADHD with medication had we restricted the category solely to those taking licensed drugs. These children tended to be older and male. Finally, we examined report of learning problems based on the question “Has a representative from a school or a health professional ever told you that you have/had a learning problem?” Among the 60% of the C8 Health Project population that gave permission to use identifying information allowing us to determine precisely who completed the questionnaire, 10.7% of children reported completing the questionnaires for themselves. Parents or legal guardians accounted for 98.2% of nonchild responders. As a sensitivity analysis, among the 60% of children where the respondent was identified, we compared the results for all children versus children where the parent or legal guardian completed the survey. Restriction to the subset with the parent/guardian as the named respondent had no effect on the pattern of results. The vast majority of the 682 children responding for themselves were old enough to be in high school and were likely adequate reporters of whether they had been diagnosed with ADHD. It is also possible that these children provided the preliminary information on the questionnaire themselves (name, birth date, address, and identification of who was completing the survey) and then passed the survey to a parent to answer the more involved questions on health outcomes. This change in respondent would not have been noted on the questionnaire.

Other covariates available for analysis included age (modeled as quintiles), sex, race/ethnicity (non-Hispanic white vs. other), body mass index (BMI) *z*-score based on the 2000 CDC growth charts of BMI for age (CDC 2009), and average household income (≤ $30,000 vs. > $30,000). Institutional review board approval was granted from the Mount Sinai Program for the Protection of Human Subjects.

*Statistical analysis.* Analysis was performed using SAS software (version 9.2; SAS Institute Inc., Cary, NC). Given the concern that PFCs may be hormonally active (White et al. 2011), we first examined the potential for sex to modify the association between exposure and outcome by comparing the effect estimates of stratified and unstratified models and by examining the *p*-value for the PFC–sex interaction term (Rothman and Greenland 1998). There was little evidence that sex modified the exposure–outcome association, although for PFOA there was a modest, imprecise, suggestion of a stronger association among females; we report unstratified models. We assessed the potential for age, sex, race/ethnicity, BMI *z*-score, and average household income to act as confounders by looking for associations between these covariates and the exposure, and these covariates and the outcome (Rothman and Greenland 1998). Neither BMI *z*-score nor race/ethnicity met the criteria for confounding, although we restricted analyses to 10,546 non-Hispanic white children (95.0%) to facilitate comparisons with other studies requiring adjustment for race/ethnicity. Because average household income was missing for 21.3% of participants, we compared odds ratios (ORs) adjusted for age, sex, and income with ORs adjusted for age and sex only. There was a < 10% change in ORs between the fully and partially adjusted models, so we excluded average household income from analysis, allowing us to retain a larger study population. We ran logistic regression models adjusted for age and sex to calculate the OR and 95% confidence intervals (CIs) for each PFC–outcome combination.

We also performed several secondary analyses: *a*) For all 10,546 children, we ran each model, simultaneously adjusting all PFCs for one another to account for confounding. For instance, in the model for PFOA, we adjusted for age, sex, PFOS, PFHxS, and PFNA. *b*) Among the 6,523 children permitting use of identifying information, we restricted analysis to the 2,437 children who lived in the same PFOA-exposed water district their entire life. Because PFOA exposure is directly related to residential location, this residential restriction is more likely to maintain the same relative ranking of PFOA exposure distribution over time, potentially reducing exposure misclassification, particularly if there is an early-life critical developmental window of susceptibility to PFOA. *c*) To facilitate comparisons with the NHANES population (Hoffman et al. 2010), we ran two additional analyses. We restricted analysis to the 3,571 children 12–15 years of age so that our age range was comparable to that of NHANES. We also examined the 5,262 children with PFOA exposures below the median (range, 0.6 ng/mL to < 28.2 ng/mL) to align our exposure range more closely to that of NHANES.

## Results

Of the 10,546 non-Hispanic white children 5–18 years of age in the C8 Health Project, 12.4% reported a diagnosis of ADHD and 12.1% a learning problem (Table 1). Using the stricter definition of ADHD diagnosis plus medication, 5.1% of children were counted as cases. Mean ± SD serum PFOA was 66.3 ± 106.1 ng/mL, and the median value was 28.2 ng/mL [interquartile range (IQR), 13.0–65.3 ng/mL], considerably higher than the NHANES 2003–2004 geometric mean of 3.9 ng/mL. Serum PFOS concentration was comparable to that of NHANES, whereas PFHxS and PFNA concentrations were higher in the C8 Health Project population. The correlation among all PFCs ranged from the weakest Spearman ρ = 0.11 (*p* < 0.001) between PFOA and PFNA to the strongest ρ = 0.54 (*p* < 0.001) between PFOS and PFHxS. The mean ± SD age in the population was 13.1 ± 3.8 years, and 51.6% were male. Of the 6,532 children in the subset where residential history was available, 2,437 (37.4%) lived their entire life in a single PFOA-exposed water district.

**Table 1:** Univariate characteristics of non-Hispanic white children 5–18 years of age, C8 Health Project, Mid-Ohio Valley, 2005–2006 (*n* = 10,546).

Characteristic	Measure
ADHD diagnosis	
Yes	1,303 (12.4)
No	9,243 (87.6)
ADHD diagnosis + medication	
Yes	542 (5.1)
No	10,004 (94.9)
Learning problem*a*	
Yes	1,281 (12.1)
No	9,265 (87.9)
Serum PFOA (ng/mL)	66.3 ± 106.1
Quartile 1: 0.6 to < 13.0	2,643 (25.1)
Quartile 2: 13.0 to < 28.2	2,634 (25.0)
Quartile 3: 28.2 to < 65.3	2,643 (25.1)
Quartile 4: 65.3 to 2070.6	2,626 (24.9)
Serum PFOS (ng/mL)	22.9 ± 12.5,
Quartile 1: 0.25 to < 14.8	2,597 (24.6)
Quartile 2: 14.8 to < 20.2	2,655 (25.2)
Quartile 3: 20.2 to < 27.9	2,632 (25.0)
Quartile 4: 27.8 to 202.1	2,662 (25.2)
Serum PFHxS (ng/mL)	9.3 ± 13.7
Quartile 1: 0.25 to < 2.9	2,716 (25.7)
Quartile 2: 2.9 to < 5.2	2,500 (23.7)
Quartile 3: 5.2 to < 10.1	2,689 (25.5)
Quartile 4: 10.1 to 276.4	2,641 (25.0)
Serum PFNA (ng/mL)	1.7 ± 1.0
Quartile 1: 0.25 to < 1.2	2,552 (24.2)
Quartile 2: 1.2 to < 1.5	2,387 (22.6)
Quartile 3: 1.5 to < 2.0	2,880 (27.3)
Quartile 4: 2.0 to 24.1	2,727 (25.9)
Sex	
Male	5,446 (51.6)
Female	5,100 (48.4)
Age (years)	13.1 ± 3.8
Quintile 1: 5.0 to < 9.3	2,092 (19.8)
Quintile 2: 9.3 to < 12.3	2,099 (19.9)
Quintile 3: 12.3 to < 14.7	2,116 (20.1)
Quintile 4: 14.7 to < 16.8	2,129 (20.2)
Quintile 5: 16.8 to < 19.0	2,110 (20.0)
Average household income	
≤ $30,000	3,968 (47.8)
> $30,000	4,335 (52.2)
BMI (kg/m^2^)	21.9 ± 5.5
BMI *z*-score	0.5 ± 1.3
BMI percentile	
Underweight (< 5th)	440 (4.4)
Healthy weight (5th to < 85th)	5,801 (58.6)
Overweight (85th to < 95th)	1,686 (17.0)
Obese (> 95th)	1,973 (19.9)
Single water district*b*	
Yes	2,437 (37.4)
No	4,086 (62.6)
Data are presented as *n* (%) or mean ± SD. **a**Learning problem denotes a positive response to the question “Has a representative from a school or a health professional ever told you that you have/had a learning problem?” **b**Available for identified subset only (*n* = 6,523).	

The association between serum PFOA and ADHD followed an inverted J. We observed a small increase in prevalence for the second quartile of exposure compared with the lowest, and a somewhat larger decrease for the highest versus lowest quartile (Table 2). For PFOS, we observed a small increase in prevalence of ADHD with medication for all quartiles that was most pronounced for the highest exposure group but less apparent for ADHD alone (without consideration of medication use). The strongest association between exposure and outcome was for PFHxS, with elevated ORs for quartiles 2–4 compared with the lowest quartile, ranging from 1.44 to 1.59. We found no discernable pattern for PFNA, with no association for ADHD alone and only a slightly elevated OR for the highest exposure quartile for ADHD with medication. Overall, the associations for ADHD both with and without medication use were similar, with the patterns more pronounced for the more stringent criteria, albeit with less precise CIs.

**Table 2:** Crude and adjusted*a* associations between serum PFC exposure and ADHD or learning problems*b* among children 5–18 years of age, C8 Health Project, Mid-Ohio Valley, 2005–2006 (*n* = 10,546).

ADHD diagnosis					ADHD diagnosis + medication					Learning problem*b*				
Serum PFC (ng/mL)	No. of cases	Crude OR	Adjusted OR (95% CI)*a*	No. of cases	Crude OR	Adjusted OR (95% CI)*a*	No. of cases	Crude OR	Adjusted OR (95% CI)*a*
PFOA																		
Quartile 1: 0.6 to < 13.0		327		1.00		1.00		129		1.00		1.00		326		1.00		1.00
Quartile 2: 13.0 to < 28.2		364		1.14		1.10 (0.94–1.30)		159		1.25		1.20 (0.94–1.53)		318		0.98		0.95 (0.81–1.12)
Quartile 3: 28.2 to < 65.3		337		1.04		0.98 (0.83–1.15)		146		1.14		1.04 (0.81–1.32)		330		1.01		0.98 (0.83–1.15)
Quartile 4: 65.3 to 2070.6		275		0.83		0.76 (0.64–0.90)		108		0.84		0.72 (0.55–0.94)		307		0.94		0.90 (0.76–1.06)
PFOS																		
Quartile 1: 0.25 to < 14.8		299		1.00		1.00		107		1.00		1.00		347		1.00		1.00
Quartile 2:14.8 to < 20.2		321		1.06		0.99 (0.83–1.17)		134		1.24		1.15 (0.89–1.50)		317		0.88		0.83 (0.70–0.98)
Quartile 3: 20.2 to < 27.9		318		1.06		0.96 (0.81–1.14)		137		1.28		1.14 (0.87–1.48)		288		0.80		0.74 (0.62–0.87)
Quartile 4: 27.8 to 202.1		365		1.22		1.09 (0.93–1.29)		164		1.53		1.27 (0.99–1.64)		329		0.91		0.85 (0.72–1.00)
PFHxS																		
Quartile 1: 0.25 to < 2.9		258		1.00		1.00		89		1.00		1.00		300		1.00		1.00
Quartile 2: 2.9 to < 5.2		304		1.32		1.27 (1.06–1.52)		128		1.59		1.44 (1.09–1.90)		313		1.15		1.13 (0.95–1.34)
Quartile 3: 5.2 to < 10.1		364		1.49		1.43 (1.21–1.70)		157		1.83		1.55 (1.19–2.04)		332		1.13		1.12 (0.95–1.33)
Quartile 4: 10.1 to 276.4		377		1.59		1.53 (1.29–1.83)		168		2.01		1.59 (1.21–2.08)		336		1.17		1.19 (1.00–1.41)
PFNA																		
Quartile 1: 0.25 to < 1.2		311		1.00		1.00		112		1.00		1.00		352		1.00		1.00
Quartile 2: 1.2 to < 1.5		302		1.04		1.00 (0.84–1.19)		117		1.12		1.02 (0.78–1.34)		299		0.89		0.87 (0.74–1.03)
Quartile 3: 1.5 to < 2.0		349		0.99		0.94 (0.79–1.11)		151		1.21		1.06 (0.82–1.36)		340		0.84		0.81 (0.69–0.95)
Quartile 4: 2.0 to 24.1		341		1.03		0.99 (0.84–1.18)		162		1.38		1.16 (0.90–1.49)		290		0.74		0.74 (0.62–0.87)
**a**Adjusted for age and sex. **b**Learning problem denotes a positive response to the question “Has a representative from a school or a health professional ever told you that you have/had a learning problem?”																		

We observed small reductions in ORs comparing quartiles 2–4 with the lowest quartile for PFOA, PFOS, and PFNA and reported learning problems, but no dose–response gradient (Table 2). The prevalence of a self-reported learning problem was increased for quartiles 2–4 of PFHxS exposure compared with the lowest quartile. The strongest OR compared the highest versus lowest quartiles (OR = 1.19; 95% CI, 1.00–1.41).

Simultaneous adjustment for all PFCs tended to weaken positive ORs and strengthen negative ORs for ADHD. The exception was for PFHxS, where simultaneous adjustment for all PFCs strengthened all of the observed positive associations. For instance, for ADHD with medication, the OR comparing the highest and lowest exposure groups was 1.67 (95% CI, 1.23–2.26) with simultaneous adjustment for all PFCs, compared with 1.59 (95% CI, 1.21–2.08) without the additional adjustment. Overall, the changes to the results with adjustment for all PFCs were minor and did not alter interpretation of the estimates (data not shown).

Among the children living in a single PFOA-exposed water district where presumably the current level of exposure reflects long-term patterns (*n* = 2,437), the association between PFOA and ADHD was attenuated (data not shown). For ADHD with medication, the modest increased prevalence in quartile 2 remained the same while the reduced prevalence in quartile 4 relative to the first quartile was eliminated (OR = 1.02; 95% CI, 0.60–1.73).

Among the 12- to 15-year-olds (*n* = 3,571), the patterns of association for ADHD alone and learning problems remained the same as for the full age range (Table 3). For ADHD with medication, the positive ORs were strengthened for PFOA and PFOS and weakened for PFHxS, and we observed no appreciable change for PFNA. Overall, the pattern of associations observed in the full data set was not materially altered by restriction to ages 12–15 years.

**Table 3:** Adjusted*a* associations [OR (95% CI)] between serum PFC exposure and ADHD or learning problems*b* among children 12–15 years of age, C8 Health Project, Mid-Ohio Valley, 2005–2006 (*n* = 3,571).

Serum PFC (ng/mL)	ADHD diagnosis	ADHD diagnosis + medication	Learning problem*b*
PFOA						
Quartile 1: 0.6 to < 13.0		1.00		1.00		1.00
Quartile 2: 13.0 to < 28.2		1.18 (0.91–1.53)		1.39 (0.95–2.04)		1.13 (0.86–1.47)
Quartile 3: 28.2 to < 65.3		0.93 (0.71–1.21)		1.25 (0.85–1.83)		0.93 (0.71–1.21)
Quartile 4: 65.3 to 2070.6		0.79 (0.60–1.04)		0.87 (0.58–1.32)		0.96 (0.73–1.26)
PFOS						
Quartile 1: 0.25 to < 14.8		1.00		1.00		1.00
Quartile 2:14.8 to < 20.2		0.91 (0.70–1.19)		1.40 (0.94–2.08)		0.78 (0.60–1.01)
Quartile 3: 20.2 to < 27.9		0.92 (0.71–1.21)		1.38 (0.92–2.06)		0.68 (0.52–0.89)
Quartile 4: 27.8 to 202.1		0.99 (0.76–1.30)		1.32 (0.88–1.99)		0.71 (0.54–0.93)
PFHxS						
Quartile 1: 0.25 to < 2.9		1.00		1.00		1.00
Quartile 2: 2.9 to < 5.2		1.46 (1.10–1.93)		1.32 (0.87–1.99)		1.31 (1.00–1.71)
Quartile 3: 5.2 to < 10.1		1.45 (1.10–1.91)		1.32 (0.88–1.97)		1.08 (0.82–1.43)
Quartile 4: 10.1 to 276.4		1.53 (1.15–2.04)		1.42 (0.94–2.13)		1.05 (0.79–1.40)
PFNA						
Quartile 1: 0.25 to < 1.2		1.00		1.00		1.00
Quartile 2: 1.2 to < 1.5		1.16 (0.89–1.51)		1.12 (0.76–1.64)		0.97 (0.74–1.25)
Quartile 3: 1.5 to < 2.0		0.96 (0.74–1.24)		0.99 (0.67–1.45)		0.86 (0.66–1.11)
Quartile 4: 2.0 to 24.1		1.00 (0.75–1.32)		1.15 (0.78–1.71)		0.73 (0.55–0.98)
**a**Adjusted for age (continuous) and sex. **b**Learning problem denotes a positive response to the question “Has a representative from a school or a health professional ever told you that you have/had a learning problem?”						

When restricting the analysis to children with PFOA exposures below the median (*n* = 5,262), for ADHD alone all ORs were near the null value and CIs were wide (data not shown). For ADHD with medication, we observed no reduction in prevalence for quartile 4 as we observed in the full population. Rather, the increase in prevalence observed when moving from the lowest quartile to quartile 2 (OR = 1.23; 95% CI, 0.85–1.76) remained consistent for quartile 3 (OR = 1.33; 95% CI, 0.93–1.93) and quartile 4 (OR = 1.35; 95% CI, 0.94–1.93).

## Discussion

Our sporadic positive associations between PFCs and ADHD contrast somewhat with the findings of Hoffman et al. (2010), even when we restricted to the same age range. In a sample of 571 children 12–15 years of age, the prevalence of ADHD was 8.4% (3.6% with medication use) in the Hoffman et al. (2010) population, compared with 14.3% (6.2% with medication use) in our population of 3,571 children 12–15 years of age. In addition to lower ADHD prevalence, the NHANES study also had lower median exposure levels for PFHxS and PFNA and, of course, dramatically lower exposures for PFOA. The median PFOS values were comparable between NHANES and the C8 Health Project. It is not surprising that PFNA exposure was higher in our sample (2005–2006) than in the NHANES samples (1999–2000 and 2003–2004), because PFNA exposure appears to be increasing (Calafat et al. 2007b) and we collected our data subsequent to NHANES. Additionally, we are studying a non-Hispanic white population, which also tends to have higher PFC levels than do non-Hispanic black and Mexican-American populations (Calafat et al. 2007b). Even with lower exposure and disease prevalence estimates, Hoffman et al. (2010) reported increased prevalence of ADHD with increased exposure to all PFCs, ranging from 1.15 (95% CI, 0.93–1.42) for the IQR effect for PFNA to 1.60 (95% CI, 1.10–2.31) for the IQR effect for PFOS. Although the results from these two studies are not directly comparable because we analyzed our exposure by quartiles to best characterize the observed nonlinear association, among our population of 12- to 15-year-olds the only positive association for ADHD alone was with PFHxS (e.g., highest vs. lowest quartile: OR = 1.53; 95% CI, 1.15–2.04). In NHANES, a 2.9-ng/mL (IQR) increase in PFHxS was associated with an OR of 1.19 (95% CI, 1.05–1.34). When examining ADHD with medication, however, our study also yielded a positive association for PFOS. In the subset of children with PFOA exposure below the median, an exposure range more comparable to that in NHANES, PFOA results were null for ADHD alone, but we observed a sustained positive association for ADHD with medication for quartiles 2–4 compared with the lowest quartile.

The pattern of results for PFOA and ADHD in our full population, with a small increase then more notable decrease in prevalence, is unlikely to be causal and may reflect some unmeasured correlate of PFOA exposure. The diagnosis of ADHD requires that behavioral symptoms be present in two or more settings, such as at home and at school (American Psychiatric Association 2000). If the geographic boundaries of water district (the strongest predictor of PFOA exposure) and school district (which may affect the likelihood of being diagnosed with ADHD) overlap, the characteristics of school districts may contribute to this finding. For instance, it is possible that children living in the water districts most highly exposed to PFOA may also be living in the school districts least likely to diagnose ADHD. Alternatively, children living in the less exposed water districts may attend schools that are more likely to diagnose ADHD. Prevalence of ADHD can vary by school district (Daley et al. 1998) as well as characteristics of teacher, school (Schneider and Eisenberg 2006), and clinical provider (Fulton et al. 2009). In addition to the potential for confounding by regional geography, another explanation for the inverted J-shaped association between PFOA and ADHD in this cross-sectional study is that a correlate of the disorder may affect measured exposure. If ADHD or its treatment is related to drinking less tap water, for example, which is a major influence on exposure, a spurious association might result. Additionally, the observed association could also occur if the condition or its treatment affected the uptake, metabolism, or excretion of PFOA. Without additional data, we are unable to more thoroughly examine any of these scenarios.

We found support for a small, positive association between PFC exposure and report of learning problems only for PFHxS. Serum PFNA levels were associated with a modest decrease in the prevalence of a self-reported learning problem. However, this outcome was defined nonspecifically and may indicate disabilities in listening, speaking, basic reading skills, reading comprehension, written expression, mathematical calculation, or mathematical reasoning (Pastor and Reuben 2008). Future investigations should differentiate among specific types of learning disabilities.

A major limitation of this cross-sectional investigation is the reliance on parent or self-report for measurement of the outcome, although we also included the more stringent case definition of ADHD report with medication use (Braun et al. 2006). One study found 83% agreement between parent report and physician records for accessing services for ADHD, and a kappa of 0.94 for agreement on medication use (Bussing et al. 2003). Parent report of ADHD is commonly used in research studies and national surveys. Furthermore, there is little reason to expect that report of ADHD would be differential by PFC exposure level, which would be needed to bias our findings away from the null. The laboratory-determined exposure measurement does not suffer from the same quality issues as do reported ADHD and learning problems. However, the simultaneous measurement of the exposure and reporting of the outcome allows for confounding by behaviors or physiologic changes associated with ADHD, such as moving to a different water district or altering tap water consumption after diagnosis, which would affect measured PFOA serum level and, additionally, preclude examining potential critical windows of developmental effects. To assess a potential causal role of PFCs in ADHD or learning problems, a prospective study is required. School-level information on this population may be needed to more adequately understand the pattern of association between PFOA and report of ADHD diagnosis. Additionally, data on other health and environmental risk factors, such as prenatal exposures to nicotine or alcohol, gestational age at birth, childhood exposures to toxicants such as lead and PCBs, and better socioeconomic information, would have enriched these analyses.

## Conclusion

With only two studies, each with its own limitations, research on the developmental health effects of PFCs is at a very early stage. The suggestive findings of these cross-sectional studies examining PFCs as risk factors for ADHD call for a more thorough evaluation of the association.
